# 免疫衰老背景下老年晚期NSCLC患者对PD-1/PD-L1抑制剂反应的异质性及干预策略

**DOI:** 10.3779/j.issn.1009-3419.2026.101.05

**Published:** 2026-03-20

**Authors:** Yurong HUANG, Yuan WANG, Aojiao WEI, Yulu XIONG, Mingzhi DUAN, Tongyao XU, Wenjie HE

**Affiliations:** ^1^650500 昆明，昆明医科大学; ^1^Kunming Medical University, Kunming 650500, China; ^2^650118 昆明，昆明医科大学第三附属医院，云南省肿瘤医院，北京大学肿瘤医院云南医院老年肿瘤科; ^2^Department of Geriatric Oncology, Kunming 650118, China; ^3^650118 昆明，生物治疗中心; ^3^Center for Biotherapy, Kunming 650118, China; ^4^650118 昆明，中西医结合科; ^4^Department of Integrative Chinese and Western Medicine, The Third Affiliated Hospital of Kunming Medical University, Yunnan Cancer Hospital, Peking University Cancer Hospital Yunnan, Kunming 650118, China

**Keywords:** 免疫衰老, 肺肿瘤, PD-1/PD-L1抑制剂, 个体化治疗, Immunosenescence, Lung neoplasms, PD-1/PD-L1 inhibitors, Individualized treatment

## Abstract

老年晚期非小细胞肺癌（non-small cell lung cancer, NSCLC）患者的比例逐年上升，尽管程序性死亡受体-1（programmed death protein 1, PD-1）/程序性死亡配体1（programmed death ligand 1, PD-L1）抑制剂彻底改变了晚期NSCLC的治疗格局，为患者带来生存获益，但老年人群因存在免疫衰老特征，衰老引发的胸腺退化、T细胞库多样性降低、慢性炎症削弱了免疫治疗的应答效率；加之临床试验中高龄患者入组不足、循证医学证据匮乏，导致其对PD-1/PD-L1抑制剂的治疗应答呈现显著异质性。临床研究发现，65-74岁患者从PD-1/PD-L1抑制剂单药或联合治疗中获益较明显，而≥75岁高龄人群疗效减弱，且免疫相关不良事件发生率升高。为优化老年患者治疗，基于综合老年评估结果制定个体化干预策略，主要包括低剂量免疫单药治疗、选择性联合治疗模式及靶向免疫衰老的新型疗法。未来研究应填补高龄患者临床证据空白，并推动基于免疫衰老程度和老年生理变化的精准治疗方案，以改善这一特殊人群的治疗结局。

## Abstract (en)

The proportion of elderly patients with advanced non-small cell lung cancer (NSCLC) is on the rise. While programmed death protein 1 (PD-1)/programmed death ligand 1 (PD-L1) inhibitors have revolutionized the management of advanced NSCLC and improved patient survival, but the elderly display prominent immunosenescence features. Age-associated thymic involution, diminished T-cell repertoire diversity, and chronic inflammation directly compromise the efficacy of immunotherapy. Furthermore, the underrepresentation of elderly patients in clinical trials and the paucity of high-quality evidence-based data jointly contribute to significant heterogeneity in their therapeutic responses to PD-1/PD-L1 inhibitors. While patients aged 65-74 years maintain significant survival benefits from immunotherapy - whether as monotherapy or combined regimens - those aged ≥75 years exhibit attenuated efficacy and heightened susceptibility to immune-related adverse events. To address these challenges, Comprehensive Geriatric Assessment (CGA) enables risk-adapted therapeutic strategies, guiding interventions such as adjusted-dose immunotherapy, selective combination therapies, and emerging approaches targeting senescence-associated immune dysfunction. Closing current evidence gaps, particularly for underrepresented octogenarians, remains imperative, as does advancing precision immunotherapy models founded on biological aging metrics rather than chronological age alone. Bridging these knowledge gaps will permit tailored treatment algorithms to optimize outcomes in this clinically complex population.

肺癌是全球范围内发病率和死亡率最高的恶性肿瘤之一，其中非小细胞肺癌（non-small cell lung cancer, NSCLC）约占所有肺癌病例的85%^[1]^。虽然控烟措施使中青年肺癌发病率有所下降，但老年（≥65岁）人群发病率仍增长10%^[2]^。由于老年人多伴有长期不良生活习惯、慢性呼吸系统基础疾病或与年龄相关的器官功能退化，导致不少患者在确诊时已进展至晚期，并对传统化疗的耐受性较差。尽管程序性死亡受体1（programmed death protein 1, PD-1）/程序性死亡配体1（programmed death ligand 1, PD-L1）抑制剂的推广已彻底改变了晚期NSCLC的治疗格局，然而真实世界数据^[3]^显示，37%的晚期NSCLC患者年龄≥75岁，而临床试验中该人群仅占全部入组患者的9%。因此，老年患者在临床试验中的代表性不足导致循证医学证据匮乏，亟需针对老年人群的特殊性开展深入研究。

PD-1/PD-L1抑制剂通过阻断PD-1与其配体PD-L1的相互作用，解除肿瘤细胞对T细胞的免疫抑制，从而增强免疫系统对肿瘤的攻击^[4]^。2025年我国专家共识（V级证据，中国临床肿瘤学会指南工作委员会制定）对PD-1/PD-L1抑制剂在驱动基因阴性NSCLC老年患者中的应用做出了更新推荐。新近循证证据^[5]^表明，不同年龄组的老年患者均可能从PD-1/PD-L1抑制剂治疗中获益。然而，当前PD-1/PD-L1抑制剂在老年NSCLC患者中的疗效有明显的异质性。荟萃分析^[6]^显示，相比于传统化疗，PD-1/PD-L1抑制剂在NSCLC患者中可显著延长总生存期（overall survival, OS）和无进展生存期（progression-free survival, PFS）[OS：风险比（hazard ratio, HR）=0.78，95%置信区间（confidence interval, CI）: 0.74-0.82）；PFS: HR=0.67, 95%CI: 0.60-0.75]。但在年龄≥75岁的高龄患者以及PD-L1肿瘤细胞阳性比例分数（tumor proportion score, TPS）<1%的人群中，PD-1/PD-L1抑制剂的OS改善效果并不显著。这种疗效异质性可能与年龄相关的免疫衰老促成的治疗应答模式改变有关。鉴于老年患者免疫治疗反应的显著差异使得临床决策复杂化，本综述将基于免疫衰老机制和临床特征建立精准选择策略，以优化这一特殊人群的治疗结局。

## 1 免疫衰老的分子特征及其对PD-1/PD-L1抑制剂疗效的调控机制

### 1.1 胸腺年龄依赖性退化与T细胞免疫衰老

胸腺随年龄增长发生不可逆的退化过程，主要表现为胸腺组织萎缩、皮质髓质交界区结构破坏、间质纤维化及脂肪细胞浸润增加。该过程引起胸腺微环境功能受损，直接影响到胸腺细胞的分化与生长发育。伴随胸腺功能衰退，幼稚T细胞比例降低，记忆T细胞比例增加，T细胞受体（T-cell receptor, TCR）多样性被限制，造成其难以高效识别新的或变异的抗原，这也是免疫衰老的核心标志之一，临床典型特征是抗肿瘤能力弱化和疫苗反应率降低^[7]^。同时，衰老T细胞线粒体氧化磷酸化效率下降，腺苷三磷酸（adenosine triphosphate, ATP）合成减少，进而激活腺苷酸活化蛋白激酶/哺乳动物雷帕霉素靶蛋白（adenosine monophosphate-activated protein kinase/mammalian target of rapamycin, AMPK/mTOR）通路失衡，AMPK缺陷促进糖酵解及HMGCR表达，经p38丝裂原活化蛋白激酶/糖原合成激酶3β（mitogen-activated protein kinase/glycogen synthase kinase 3β, MAPK/GSK3β）信号通路上调PD-1^[8]^；另外，在甲硫氨酸缺乏微环境中，MAPK表达降低致H3K79甲基化减少，进一步增加PD-1表达^[9]^，共同促进T细胞耗竭，引起免疫功能紊乱，降低老年患者对PD-1/PD-L1抑制剂的响应效率。此外，在CD8^+^幼稚T细胞中，转录因子复合物的组成发生显著失调，而CD4^+^幼稚T细胞的复合物库则保持相对稳定的状态，这种差异或许是老年患者免疫应答存在异质性的关键基础^[10]^。近期已通过多中心人类队列（如训练集n=392）建立的基于幼稚T细胞的免疫年龄预测模型属于临床验证型预测工具，通过探究CD38表达水平的动态变化以及TCR多样性，估算胸腺输出功能与免疫衰老程度^[11]^。

### 1.2 衰老相关免疫抑制微环境的特征与机制

炎性衰老是衰老细胞通过衰老相关分泌表型（senescence associated secretory phenotype, SASP）释放白细胞介素（interleukin, IL）、趋化因子、蛋白酶和生长因子等多种促炎因子，它首先通过旁分泌机制诱导周围细胞进入衰老状态，从而在组织微环境内产生持续的炎症刺激和信号放大效应，该现象往往会促进肿瘤转移和心血管等退行性病变的发展。此外，在衰老个体尤其是肿瘤患者中，IL-6等分泌因子通过Janus激酶/信号转导与转录激活因子3（Janus kinase/signal transducer and activator of transcription 3, JAK/STAT3）通路诱导DNA甲基转移酶1（DNA methyltransferase 1, DNMT1）表达，导致PD-L1启动子区去甲基化，从而增强其转录活性。这一过程促使肿瘤细胞上调PD-L1表达，并促进调节性T细胞（regulatory T cells, Tregs）和骨髓源性抑制细胞（myeloid derived suppressor cells, MDSCs）等免疫抑制细胞的募集，这一过程导致衰老相关免疫功能障碍及免疫抑制肿瘤微环境的形成，最终致使老年人对PD-1/PD-L1抑制剂的反应率显著降低^[12]^。这种衰老相关的免疫调控失衡不仅削弱了T细胞的抗肿瘤功能，还导致PD-1/PD-L1抑制剂在老年患者中的临床反应率显著降低。该机制揭示了衰老、表观遗传调控和免疫逃逸之间的复杂关联，为优化老年肿瘤患者的免疫治疗策略提供了理论依据。

综上，免疫衰老与炎性衰老在分子机制和临床表现上既有明显区别又存在密切联系。胸腺退化是免疫衰老的核心特征之一，其通过下调FOXN1表达影响AIRE等分子的功能，导致T细胞阳性选择受损、TCR多样性降低，并促使CD8^+^ TEMRA细胞的克隆扩增及功能耗竭，最终削弱对新抗原的免疫应答能力。与此同时，炎性衰老则通过SASP分泌促炎因子（如IL-6），刺激STAT3等信号通路上调PD-L1表达，塑造肿瘤免疫抵抗微环境。二者通过“胸腺-外周-微环境”的三级调控网络实现级联放大（图1），慢性炎症通过促炎因子加速免疫细胞衰老，而免疫衰老又进一步削弱炎症调控能力，形成恶性循环，最终构成了老年人群免疫治疗异质性的分子基础。

**图1:**
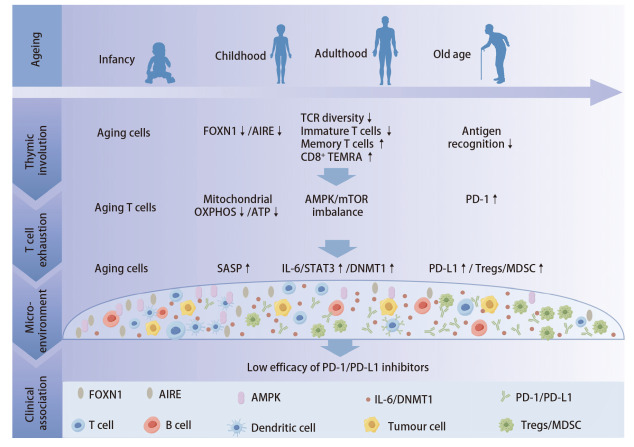
免疫衰老调控PD-1/PD-L1抑制剂反应的机制图，主要包括胸腺退化、T细胞耗竭、免疫抑制微环境3个模块。

## 2 疗效与安全性的年龄异质性：从临床研究到真实世界

### 2.1 PD-1/PD-L1抑制剂单药治疗的年龄差异

PD-1/PD-L1抑制剂在老年NSCLC患者中的疗效存在显著人群异质性。在PD-L1高表达（TPS≥50%/免疫细胞阳性比例≥50%/浸润免疫细胞占比≥10%）的晚期NSCLC患者中，帕博利珠单抗、阿替利珠单抗等PD-1/PD-L1抑制剂单药治疗已被KEYNOTE-024^[13]^、IMpower110^[14]^等III期临床试验（I级证据，欧洲肿瘤内科学会临床获益程度量表2.0标准）验证为有效替代治疗方案，帕博利珠单抗组患者的OS获益HR为0.64（95%CI: 0.42-0.98），阿替利珠单抗组的OS获益HR为0.78（95%CI: 0.45-1.36）。然而临床试验通常纳入经过严格筛选的健康老年患者[美国东部肿瘤协作组体力状态（Eastern Cooperative Oncology Group performance status, ECOG PS）评分0-1分]，真实世界患者更异质化。一项回顾性研究^[15]^发现，接受单药帕博利珠单抗或铂类/培美曲塞/帕博利珠单抗联合治疗的患者中位OS（11.4、12.9个月）显著短于KEYNOTE-024^[13]^（26个月）和KEYNOTE-189^[16]^（22.8个月）临床试验的结果，部分原因可能是真实世界患者更虚弱或合并症负担更重。此外，研究^[17]^发现老年晚期癌症患者接受免疫治疗的OS、PFS存在年龄依赖性下降趋势，但这种差异在调整PS和基础并发症等混杂因素后部分减弱。这说明年龄并非是疗效差异的唯一因素，伴随年龄增长出现的机体功能衰退和合并症负担也是影响疗效的主导因素。这一发现具有重要的临床指导价值，提示临床医生在为老年患者制定免疫治疗决策时，不能仅依据实际年龄，而应全面考察患者的PS和共病情况，以更准确地预测治疗获益。

### 2.2 PD-1/PD-L1抑制剂联合治疗的年龄差异

RATIONALE-307^[18]^、KEYNOTE-189^[16]^、CHOICE-01^[19]^等多项III期临床研究结果显示（IA级证据，欧洲肿瘤内科学会临床获益程度量表2.0标准），对于年龄65-74岁、PS可耐受化疗的患者，替雷利珠单抗、帕博利珠单抗、特瑞普利单抗等PD-1/PD-L1抑制剂联合化疗的治疗方案在PFS方面展现一致获益（HR=0.52-0.64）。尽管上述研究的OS数据尚未完全成熟，但目前多数结果显示OS呈现改善趋势（HR=0.64-0.78）。基于此，对于此类老年患者，若无禁忌证，应优先考虑PD-1/PD-L1抑制剂联合含铂双药化疗的一线治疗方案。尽管PD-1/PD-L1抑制剂联合化疗在低龄老人晚期NSCLC患者中显示出明确的生存获益，但这一优势在≥75岁老年患者中存在争议。该联合方案在这一高龄人群未能显著改善OS，反而可能增加≥3级免疫相关不良事件（immune related adverse events, irAEs）的发生风险。纳武利尤单抗联合化疗的回顾性分析^[20]^显示，联合治疗方案虽可适度延长老年患者的中位OS（18.8 vs 15.6个月，HR=0.86，95%CI: 0.69-1.08，P=0.1859），但尚未达到统计学显著差异，且严重治疗相关AEs的发生率明显增加。再进一步针对PS较差的特定亚组分析时发现，ECOG PS为2分的患者，联合治疗方案的生存获益可能不及传统化疗方案。荟萃分析^[6]^进一步证实，随着患者年龄增长，PD-1/PD-L1抑制剂的疗效可能逐渐减弱，而老年人群的irAEs管理也面临更大挑战，在年龄≥75岁且PD-L1 TPS<1%的亚组中未观察到OS显著延长，ECOG PS评分≥2分的患者获益也有限。这些相矛盾的研究结果表明，不同年龄的分层方式（如65-74 vs ≥75岁）中，老年晚期NSCLC患者对PD-1/PD-L1抑制剂的治疗应答有显著差异，背后机制或许跟免疫系统的渐进式老化过程密切相关^[21]^。如前文所述，胸腺萎缩、T细胞功能缺陷及持续性低度慢性炎症等特征的免疫衰老，可能通过抑制肿瘤免疫应答而削弱PD-1/PD-L1抑制剂在高龄NSCLC患者中的疗效。

### 2.3 irAEs的年龄异质性

关于老年癌症患者irAEs的流行病学特征，现有研究也呈现出一定差异。前瞻性临床研究如CheckMate-153^[22]^的亚组分析显示，70岁以上的老年患者中有39%的患者在接受纳武利尤单抗治疗后，所有级别AEs（约60%）及严重治疗相关AEs（约6%）与非老年人群相当。然而多项真实世界研究表明，老年患者irAEs可能呈现轻度升高趋势。一项基于美国食品药品监督管理局AEs报告系统数据库（2015-2023年）的大规模回顾性分析^[23]^显示，65-74和75-84岁患者的irAEs总体风险显著高于18-64岁人群。此外，irAEs谱呈现显著年龄异质性，心血管irAEs随年龄增长而上升（75-84岁达峰值），内分泌irAEs则逐渐下降，而肝胆、胃肠道和眼部irAEs减少，肾脏及肌肉骨骼irAEs风险增高。虽然各真实世界研究结论存在不一致性，多数证据表明老年与非老年患者irAEs谱整体相似但前者存在升高趋势，这种差异可能与衰老相关的免疫稳态改变有关，不同器官的免疫微环境衰老程度不同，可能解释irAEs的器官分布差异。在老年患者群体中，临床特别需要关注老年患者免疫治疗相关性肺炎、结肠炎和皮肤毒性等常见irAEs。肺炎的危害性在于老年患者肺功能储备下降且常合并基础肺部疾病，免疫介导的肺损伤易被误诊为感染，导致治疗延误和较高死亡率；老年群体患结肠炎时，易出现严重的水电解质紊乱，随后引发后续心血管、肾功能失代偿，治疗采用的激素和免疫抑制剂会引发基础病恶化；皮肤毒性虽看似轻微，但可能直接影响生活质量并预示更严重毒性，且早期皮肤反应可作为后续irAEs的预警信号。这三类AEs发病频次高、潜在隐患大，属于目前临床监测的核心要点。而中国NSCLC患者免疫检查点抑制剂相关肺炎（immune checkpoint inhibitor-associated pneumonitis, ICI-P）风险增高可能与亚洲人群肺纤维化高发及PD-L1/人类白细胞抗原I（human leukocyte antigen class I, HLA-I）共表达失衡导致的免疫耐受失调有关^[24]^。在老年患者的治疗管理上，需权衡整体健康状况与irAEs严重程度来调整治疗方案，并实施密切监测。

综上，现行临床试验的高龄老年患者入组不足，也可能部分解释真实世界中的免疫治疗效果异质性（表1）。伴随年龄增长，机体功能储备下降、多病共存及免疫衰老不仅削弱抗肿瘤应答，还可能加剧irAEs的发生风险。

**表1:** 按年龄分层（65-74 vs ≥75岁）汇总PD-1/PD-L1抑制剂在老年晚期NSCLC患者中的疗效与安全性数据

Clinical trial (NCT ID)	Trial design	Intervention	Number of cases	OS (HR, 95%CI)	≥grade 3 irAEs
CheckMate 017 (NCT01642004)^[25]^	Phase III, later-line	Nivo vs Doc	65-74 yr: 91 (33.5%) ≥75 yr: 29 (10.7%)	65-74 yr: 0.56 (0.34-0.91) ≥75 yr: 1.09 (0.58-2.05)	Nivo: 6% Doc: 52%
CheckMate 057 (NCT01673867)^[26]^	Phase III, later-line	Nivo vs Doc	65-74 yr: 200 (34.4%) ≥75 yr: 43 (7.4%)	65-74 yr: 0.63 (0.45-0.89) ≥75 yr: 0.90 (0.43-1.87)	Nivo: 9% Doc: 53%
CheckMate 227 (NCT02477826)^[27]^	Phase III, first-line	Nivo+Ipi vs Platinum chemo	65-74 yr: 157 (39.6%) ≥75 yr: 40 (10.1%)	65-74 yr: 0.75 (0.61-0.92) ≥75 yr: 0.85 (0.56-1.28)	Nivo+Ipi: 32.8% Chemo: 36%
CheckMate 9LA (NCT03215706)^[28]^	Phase III, first-line	Nivo+Ipi+2×Platinum chemo vs 4×Platinum chemo	65-74 yr: 148 (41%) ≥75 yr: 37 (10%)	65-74 yr: 0.62 (0.46-0.85) ≥75 yr: 1.21 (0.69-2.12)	NR
IMpower150 (NCT02366143)^[29]^	Phase III, first-line	Atezo±Bev+Carbo/ Paclitaxel vs Bev+Platinum chemo	65-74 yr: 149 (37.3%) ≥75 yr: 36 (9.1%)	65-74 yr: 0.76 (0.56-1.00) ≥75 yr: 0.78 (0.51-1.19)	NR
IMpower110 (NCT02409342)^[30]^	Phase III, first-line	Atezo vs Platinum chemo	65-74 yr: 80 (39%) ≥75 yr: 23 (11.2%)	65-74 yr: 0.78 (0.45-1.36) ≥75 yr: 1.03 (0.31-3.48)	NR

yr: years; OS: overall survival; HR: hazard ratio; CI: confidence interval; irAEs: immune-related adverse events; NSCLC: non-small cell lung cancer; Nivo: Nivolumab; Doc: Docetaxel; Ipi: Ipilimumab; Atezo: Atezolizumab; Bev: Bevacizumab; Carbo: Carboplatin; chemo: chemotherapy; Platinum chemo: Platinum-based chemotherapy; NR: not reported

## 3 干预策略及优化治疗方案

### 3.1 综合老年评估的预测价值

老年患者所特有的免疫衰老现象以及PS的差异，使传统的ECOG PS在评估整体健康状况时存在明显局限，无法高效指导临床诊疗决策的实施。综合老年评估（Comprehensive Geriatric Assessment, CGA）对疾病状态、精神心理、躯体功能和社会支持等多个维度的因素开展全面评估，可以察觉潜在的健康问题，进而指导制定具有针对性的干预措施，以此优化治疗结果并提高生活水准^[31]^。尽管CGA最初并非针对肿瘤患者设计，但其评估工具和相关量表已广泛应用于老年肿瘤患者的临床管理。国际通用的老年衰弱筛查工具G8量表（Geriatric 8）囊括8个评估项目，涉及活动能力、认知功能、营养状况、心理境况及合并用药等方面，总分0-17分，评分≤14分提示患者存在衰弱风险。G8量表因所需时间短且经济成本较低适合门诊预筛，于临床诊疗实践中，推荐对G8评分>14分的患者实施常规诊疗，而对于评分≤14分的衰弱患者，进一步运用美国临床肿瘤学会推荐的老年评估工具开展全面评估并进行针对性干预。前瞻性研究（如ELDERS研究^[32]^）证实，在老年患者接受PD-1/PD-L1抑制剂治疗时，运用G8量表等筛查工具与CGA配合可有效识别虚弱状态，进而预测3-5级irAEs的发生率，如ECOG PS评分≥2分或G8≤14分时启动减量治疗。

免疫治疗中CGA应用受关注程度日益增加，多项研究对其核心评估工具的临床应用价值给予支持，日常生活能力（Activities of Daily Living, ADL）评估所得结果，既可以体现患者功能障碍程度，还与免疫衰老标志物（如CD8^+^/CD4^+^比例增高等现象）密切关联。慢性巨细胞病毒感染的患者ADL评分每减少1分，irAEs的发生率会攀升12%，这显示ADL评估在识别高风险人群方面有预警作用^[33]^。当涉及神经认知功能评估时，简易精神状态检查（Mini-Mental State Examination, MMSE）及其数字化的改良形式已用于监测免疫治疗造成的认知功能障碍，特别是在远程随访和多中心认知协作研究中彰显了便捷的好处^[34]^。高级认知功能的精细评估则需要依赖更敏感的工具，改良型工具性ADL量表（Instrumental ADL, IADL）如阿姆斯特丹问卷可捕捉免疫抑制剂对复杂认知功能（如财务管理、服药管理）的潜在影响，相较传统ADL量表具有更优的信效度^[35]^。然而，当前多数研究为观察性或小样本分析，缺乏针对CGA指导PD-1/PD-L1抑制剂治疗的随机对照试验。综上，现有的证据说明CGA在老年肺癌患者PD-1/PD-L1抑制剂治疗中存在潜在应用价值，尤其是在毒性风险分级与个性化治疗方案的选择上，但该临床适用性仍要更多高质量研究进行验证。

### 3.2 治疗方案调整

#### 3.2.1 基于免疫衰老程度的剂量调整策略

老年晚期NSCLC患者的免疫衰老状态显著影响PD-1/PD-L1抑制剂的疗效与安全性。根据一项高质量的Cochrane系统评价^[36]^，高龄老年患者对PD-1/PD-L1抑制剂单药的耐受性优于联合化疗方案，且单药治疗可提供相似的生存获益，单药PD-1/PD-L1抑制剂可改善OS（HR=0.68, 95%CI: 0.60-0.76）、PFS（HR=0.68, 95%CI: 0.52-0.88），且AEs发生率可能较低，相对危险度（relative risk, RR）为0.41（95%CI: 0.33-0.50），异质性（heterogeneity, I²）为62%。这与免疫衰老背景下联合治疗的毒性增加有关，尤其是化疗联合PD-1/PD-L1抑制剂时心律失常和高血压风险显著升高。Sun等^[37]^发现，对高龄及PS较差的群体，低剂量方案（PD-1抑制剂120或40或100 mg/3周）与标准剂量（帕博利珠单抗200 mg或纳武利尤单抗240 mg/3周）相比，可维持70%以上PD-1受体占有率并保持疗效，同时降低irAEs。数据提示特殊人群采用低剂量免疫治疗可实现最佳风险获益平衡（II-III类证据，欧洲肿瘤内科学会推荐分级推荐）。此外，真实世界研究^[38]^发现，帕博利珠单抗延长间隔给药方案（400 mg/6周）与标准间隔方案（200 mg/3周）疗效相当，提示未来老年患者接受PD-1/PD-L1抑制剂单药治疗时需密切监测药代动力学参数与毒性反应，优化给药间隔以平衡疗效与安全性。

#### 3.2.2 联合治疗模式

基于现有证据，晚期NSCLC治疗决策需综合年龄、PD-L1表达、驱动基因状态及耐受性制定个体化免疫联合策略。对于年龄为65-74岁或PD-L1 TPS<50%的晚期NSCLC患者，可以耐受者优先推荐PD-1/PD-L1抑制剂联合含铂双药化疗（I类推荐，美国国立综合癌症网络2025年第7版）^[5]^。化疗药物在联合治疗中不仅发挥直接的细胞毒作用，还可通过上调肿瘤细胞PD-L1表达、增强T细胞毒性、释放肿瘤抗原以及减少免疫抑制性Tregs等方式改善肿瘤免疫微环境，从而协同增强PD-1/PD-L1抑制剂的疗效^[39]^。而抗血管生成药物联合免疫治疗的机制在于靶向血管内皮生长因子（vascular endothelial growth factor, VEGF）/VEGF受体（VEGF receptor, VEGFR）信号通路改善肿瘤微环境，包括减轻组织缺氧、促进CD8^+^ T细胞浸润以及减少肿瘤相关巨噬细胞的募集，从而增强PD-1/PD-L1抑制剂的免疫治疗效果^[40]^。回顾性分析数据^[41]^表明，联合抗血管生成药物治疗组与化疗组相比，疾病控制率（disease control rate, DCR）显著提高（96.3% vs 58.3%, P=0.001），且这种获益不受PD-L1表达水平的限制，同时证实联合治疗的整体安全性可控。值得注意的是，抗VEGF药物可能通过保护血管完整性降低肺炎风险，这为高风险亚洲患者提供了潜在干预靶点^[42]^。尽管抗血管生成药物联合免疫治疗在晚期NSCLC中显示出优于化疗的效果，但老年患者往往会出现血管内皮功能老化，这会对抗血管生成药物的耐受程度造成影响^[43]^，且目前已公布的临床数据尚缺乏明确的年龄亚组分析来指导精准决策。此外，放疗通过重编程肿瘤免疫微环境（如诱导自噬）增强免疫治疗效果，尤其适用于局部进展的患者，但老年患者可能存在放射诱导免疫激活减弱的生物学特性^[44]^。Xu等^[45]^开展的研究结果表明，在免疫治疗前3个月内接受放疗的患者OS明显长于未接受放疗的患者，中位OS延长3.1个月（15.3 vs 12.2个月）。目前联合策略主要采用序贯或同步方案，但需特别注意治疗时机选择。

需警惕的是，尽管双重免疫检查点阻断（如PD-1联合细胞毒性T淋巴细胞相关抗原4抑制剂）能协同激活肿瘤微环境中的免疫应答机制，但显著增加irAEs发生率，且目前尚缺乏可靠的生物标志物来精准筛选潜在获益人群^[46]^。此外，只有20%的表皮生长因子受体（epidermal growth factor receptor, EGFR）突变型NSCLC患者可从免疫疗法中获益^[47]^，这种差异在老年患者中可能更为明显。对于高龄患者，目前缺乏足够的循证依据支持常规使用靶向-免疫联合方案，需综合评估免疫衰老和合并症风险，个体化选择治疗策略。

### 3.3 基于免疫衰老的新兴疗法

就免疫系统而言，通过IL-7或生长激素促进胸腺再生、采取清除衰老细胞等靶向免疫器官和衰老免疫细胞的策略，可有效恢复年龄相关的免疫功能衰退情况^[48]^。采用IL-7和PD-1抑制剂联合治疗荷瘤小鼠，可极大提高肿瘤浸润CD8^+^效应T细胞的比例，减少Tregs与耗竭性T细胞的数量，其抗肿瘤效果显著优于单一疗法^[49]^；生长激素做预处理后与PD-1抑制剂联合，可以恢复老年荷瘤小鼠的胸腺功能，增强机体对PD-1抑制剂的响应灵敏度，延长小鼠的OS^[50]^，提示靶向免疫衰老的联合策略具有临床转化潜力。同时，达沙替尼联合槲皮素作为衰老细胞清除剂，通过靶向清除p16INK4a^+^衰老细胞，减少SASP分泌，极大改善肿瘤免疫抑制微环境，在晚期实体瘤患者所进行的I期临床试验（NCT04313634）^[51]^中，该联合治疗方式的安全性良好，还使部分之前对PD-1无响应的患者取得显著临床益处。此外，调控核因子-κB（nuclear factor-κB, NF-κB）/mTOR等促衰老信号通路或激活AMPK/沉默信息调节因子1（sirtuin 1, SIRT1）等抗衰老通路，可降低慢性炎症程度并改善免疫微环境^[52]^。然而上述疗法多处于临床前研究或I期临床试验阶段，还面临诸多转化医学挑战，如以IL-7为核心的联合治疗可能加重PD-1抑制剂相关毒性（如细胞因子风暴）^[53]^，缺乏衰老特异性指标的评估体系，尚无基于衰老程度分层的个体化给药方案，同时药物安全性、作用靶点特异性及规模化生产等问题也亟待解决。前期研究数据虽显示出潜在的临床价值，但其确切疗效与安全性仍需通过大规模II/III期随机对照试验加以验证。

综上，老年NSCLC患者的免疫治疗需充分考虑免疫衰老和治疗耐受性特点。CGA在筛选免疫治疗候选人群方面显示出重要价值，但依旧需要更高质量的证据作支撑。基于年龄的分层策略显示，高龄（≥75岁）患者也许更适合免疫治疗单药或减量的治疗方案，而低龄老年患者（65-74岁）若身体条件允许，可能会从联合治疗方案中受益。这些多靶点、多层次的干预策略共同组建了针对免疫衰老相关肿瘤的综合治疗体系，为促进老年癌症患者的治疗预后开拓了新途径。

## 4 总结与展望

免疫衰老背景状态下，老年晚期NSCLC患者的治疗面临严峻挑战，尽管PD-1/PD-L1抑制剂极大改变了晚期NSCLC治疗格局，但在高龄患者中其疗效表现出明显的异质性。临床研究显示，≥75岁患者相较年轻人群获益有限，且irAEs的增加往往影响治疗方案的持续性，这可能与衰老引发的胸腺退化、T细胞功能衰竭及慢性低度炎症状态下微环境改变相关。这些问题在真实世界临床实践中更为突出，≥75岁的高龄患者多为临床试验的排除人群，研究中PS较好的入选偏倚会使报告中理想数据与临床常规诊疗差距增加。此外，高龄造成的多重复杂问题，不能仅靠年龄时间点的分割去划定疗法，所伴随的心肺血管、代谢等全身状态，还有各器官储备力量的衰退，均为潜在决定性影响因素。

我国老年NSCLC患者具有显著的病因学与临床治疗特殊性：首先是合并疾病谱与欧美人群存在差异，慢性乙型病毒性肝炎（hepatitis B virus, HBV）感染的普遍性使其在接受免疫治疗时存在较高的病毒再激活风险，可能导致严重的免疫相关性肝炎，该风险在真实世界研究中已得到印证但仍缺乏系统性评估证据；其次，中国人特有的CYP2C19慢代谢基因型（>35%）常导致免疫治疗药物代谢异常，而广谱抗生素和传统中药可能通过调控肠道菌群和竞争性抑制CYP450酶系统进一步影响药效和安全性，但目前老年患者的群体药代动力学研究仍属空白；最后多病共存导致多重用药现象显著，60%的患者同时用5种以上药物，繁杂的基础病用药进一步增加了药物转化代谢的动态不定性，或许会放大免疫治疗的毒性效应，在中国特色治疗管理方案里，需系统性考量这些综合因素。针对这一临床异质现象，现有的干预手段正朝着更个体化的方向前行。临床上极为迫切地需要采用CGA构建患者的系统临床画像，这能够精细区分患者的实际生物学年龄，继而匹配恰当的治疗强度。治疗相关的灵活调整正逐步形成共识：剂量减停方案能更有效地平衡高龄患者的安全获益关系；免疫联合治疗的模式借助多重机制增强疗效表现；且以免疫衰老为关键靶点的新型治疗举措，有望为免疫治疗响应不佳的高龄肺癌患者带来新的治疗出路。

综上所述，现有文献中直接针对中国人群的分子特征研究仍有限，中国老年肺癌免疫治疗研究需重点关注人群特异性问题与转化医学突破。首先，针对中国老年NSCLC患者（尤其≥75岁群体）开展多中心真实世界研究，同时允许轻度共病患者参与到试验中，通过前瞻性干预研究系统评估合并基础肺病及EGFR突变状态对免疫治疗安全性的影响，需开发针对老年群体的疗效评价指标体系。其次，探索胸腺再生疗法（如低剂量IL-7）联合PD-1抑制剂的临床转化价值，并通过多组学方法（如单细胞测序、TCR库分析等）建立涵盖免疫衰老特征的中国老年人群特异性生物标志物预测体系，重点纳入中国人群高发的HBV感染背景和特殊饮食模式（如高脂低蛋白）等因素对免疫重建的影响。第三，建立基于CGA的前瞻性研究以指导个体化治疗策略，重点验证衰弱量表指导的剂量调整方案。更关键的发展核心是建立依托老年特异性药代动力学特征的中国特色精准给药模型：需要考虑原发性肺结核病灶或HBV所产生的免疫记忆细胞的功能状态；兼顾患者长期服用的中医药成分可能对多种核受体易位产生的干预影响；准确把握具有老年特征的消化道菌群状况导致的免疫调节异常，最终通过机器学习算法整合上述三维指标，建立“病因学-药物相互作用-生理储备”导向的精准给药模型，让免疫治疗的剂量调整有据可依。展望未来管理策略，基于中国老年患者的特殊临床背景，有必要系统性构建具有地域特色的免疫治疗决策路径。首先依托中国人群特有遗传背景，整合基因组学、表观组学、免疫组学等多组学数据，开发可实现免疫衰老程度量化的预测模型；其次搭建与中国医疗资源分布相匹配的监测体系，参考相关“中国方案”提出的诊疗流程^[54]^，构建从生物标志物检测到治疗方案选择的完整决策体系，最终实现老年患者的个性化精准治疗干预。
